# Statistical design and analysis in trials of proportionate interventions: a systematic review

**DOI:** 10.1186/s13063-019-3206-x

**Published:** 2019-02-28

**Authors:** Jane Candlish, M. Dawn Teare, Judith Cohen, Tracey Bywater

**Affiliations:** 1 ScHARR, University of Sheffield, 30 Regent Court, Sheffield, S1 4DA UK; 2 Department of Health Sciences, University of York, Area 2, Seebohm, Rowntree Building, York, Y010 5DD UK; 3 Hull Health Trials Unit, University of Hull/Hull York Medical School, York, UK

**Keywords:** Systematic review, Complex intervention, Trial, Proportionate intervention, Stepped care, Adaptive treatment strategy, Adaptive intervention, Sequential multiple assignment randomised trial, Proportionate universalism

## Abstract

**Background:**

In proportionate or adaptive interventions, the dose or intensity can be adjusted based on individual need at predefined decision stages during the delivery of the intervention. The development of such interventions may require an evaluation of the effectiveness of the individual stages in addition to the whole intervention. However, evaluating individual stages of an intervention has various challenges, particularly the statistical design and analysis. This review aimed to identify the use of trials of proportionate interventions and how they are being designed and analysed in current practice.

**Methods:**

We searched MEDLINE, Web of Science and PsycINFO for articles published between 2010 and 2015 inclusive. We considered trials of proportionate interventions in all fields of research. For each trial, its aims, design and analysis were extracted. The data synthesis was conducted using summary statistics and a narrative format.

**Results:**

Our review identified 44 proportionate intervention trials, comprising 28 trial results, 13 protocols and three secondary analyses. These were mostly described as stepped care (*n*=37) and mainly focussed on mental health research (*n*=30). The other studies were aimed at finding an optimal adaptive treatment strategy (*n*=7) in a variety of therapeutic areas. Further terminology used included adaptive intervention, staged intervention, sequentially multiple assignment trial or a two-phase design. The median number of decision stages in the interventions was two and only one study explicitly evaluated the effect of the individual stages.

**Conclusions:**

Trials of proportionate staged interventions are being used predominantly within the mental health field. However, few studies consider the different stages of the interventions, either at the design or the analysis phase, and how they may interact with one another. There is a need for further guidance on the design, analyses and reporting across trials of proportionate interventions.

**Trial registration:**

Prospero, CRD42016033781. Registered on 2 February 2016.

**Electronic supplementary material:**

The online version of this article (10.1186/s13063-019-3206-x) contains supplementary material, which is available to authorized users.

## Background

Many health, educational and social interventions have multiple components. For a proportionate intervention, these complex multi-component interventions are delivered in a proportionate or adaptive manner, in which the components of the intervention are delivered in response to an individual’s need over time. Other analogous terms for a proportionate intervention include adaptive intervention, dynamic treatment regime and stepped care.

Proportionate interventions are multi-stage and defined by a series of decision rules. The key features are critical time points, tailoring variables and treatment options. An example of an adaptive intervention was described by Almirall et al. [1]. This intervention aimed to improve outcomes for children with autism who had minimal verbal skills. The treatment was discrete teaching and was delivered at stage 1 as two sessions per week over 12 weeks. At 12 weeks, the child was assessed for change in spontaneous communication utterances since baseline (the tailoring variable). At the critical time point at 12 weeks, if the spontaneous communication utterances increased by at least 25%, then the discrete teaching treatment continued at two sessions per week for the next 12 weeks. If the change was below this, then the discrete teaching treatment increased to three sessions per week.

The proportionate approach is based on the notion that individuals differ in their response to treatment. Individuals who require a step-up, step-down or switch in intervention receive it. For those who are responding to the current intervention, there is no increase in burden, such as side effects or invested time. Additionally, all interventions incur costs and multi-component interventions can be both costly and resource intensive. Providing treatment appropriate to individual need should improve efficiency by reducing the costs of unnecessary further treatments whilst conserving resources for those in greatest need. Proportionate interventions are in keeping with recommendations from the Strategic Review of Health Inequalities in England after the 2010 Marmot Review [2], which stated that actions and interventions should be both universal and targeted to reflect the level of need or disadvantage. This idea was termed ‘proportionate universalism’. Recent advances include just-in-time adaptive interventions, which deliver treatment sensitive to an individual’s changing needs for support. With technology, treatment can be based on measurements of rapidly changing factors. In health or behavioural change interventions, this allows a treatment to be delivered when a person is (a) vulnerable or open to positive changes and (b) receptive. Nahum-Shani et al. [3] developed a conceptual framework to help guide the development of just-in-time adaptive interventions, which is likely to be used more commonly in clinical trials in the near future.

Evaluating a proportionate intervention in a randomised controlled trial presents fresh challenges for the statistical study design and analysis outside current guidelines for complex intervention research [4, 5]. Teams developing such proportionate interventions may wish to optimise the intervention and thus, may want to evaluate the incremental effectiveness of the individual stages in addition to the overall intervention. In general, trials randomise individuals or clusters to a whole intervention package to assess effectiveness. However, a proportionate intervention creates a variable number of levels of intervention and frequently multiple hierarchical levels of clustering occur, each dependent upon outcomes at the previous stage of intervention. Clustering may be non-random and dependent on an intermediate outcome.

This review aimed to identify trials of proportionate interventions and how they are being designed and analysed in current practice. Research into proportionate and adaptive interventions has previously been done in other forms or with a slightly different focus to this review [3, 6]. Early work by Collins et al. [6] presents a conceptual framework for adaptive interventions. They discuss key design principles including choice of tailoring variables and the derivation of good decision rules. A good decision rule is objective and comprehensive and it will ensure intervention components are delivered to individuals at the intended intensity. Nahum et al. [3] reviewed how adaptive interventions use decision rules to link individual responses with intervention options and the repeated use of these rules to adapt interventions over time in response to the changing response of individuals. They discuss how sequential multiple-assignment randomised trials (SMARTs) can be used to construct adaptive interventions, using a case study of an adaptive intervention for children with attention deficit hyperactivity disorder (ADHD) to illustrate analysis methods for the SMART design. They compare first- and second-stage intervention options, and the interventions embedded within the SMART design. Proportionate or adaptive interventions are desirable due to the heterogeneous responses to treatments. Some people may need only a low-intensity intervention while others may need a higher intensity or an alternative intervention. However, the design of adaptive intervention strategies must be driven by the scientific research question. Almirall et al. [1] present an informative review of the optimal design and evaluation of adaptive interventions in education research.

The current review moves beyond the work that has already been conducted. It systematically reviews the methods used in studies of proportionate interventions. It will be useful for those planning and analysing trials in this area, since we present fields in which proportionate interventions are currently being utilised, the types of tailoring variables and the decision rules used.

We conducted a systematic review of published trials to present the types of proportionate interventions being evaluated and the design and analysis methods being undertaken in current practice. Without knowing the variety of proportionate interventions and scenarios that exist, methodological work investigating suitable design and analysis strategies cannot be focused appropriately. The specific objectives of this systematic review were to:

1. Explore how trials of proportionate interventions are being designed in practice
2. Review the type of statistical design and analysis methods being implemented in trials involving staged proportionate interventions
3. Review whether trials of proportionate interventions are being analysed differently to trials of non-proportional interventions and if the component parts are considered in the analysis.

## Methods

Details of the protocol for this systematic review were registered on PROSPERO (CRD42016033781). We conducted the review according to the Preferred Reporting Items for Systematic Reviews and Meta-Analyses (PRISMA) guidelines for reporting systematic reviews where relevant [7]. A completed PRISMA checklist [7] is available as Additional file 1.

### Literature search

**Fig. 1:**
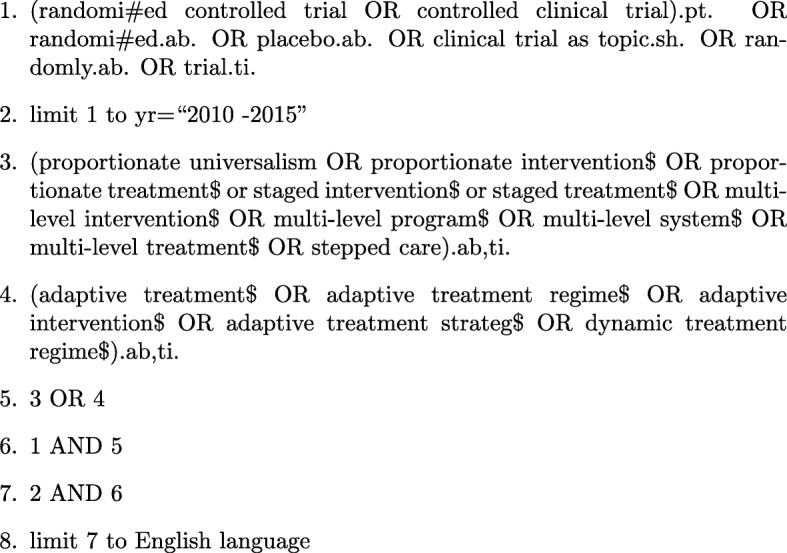
MEDLINE search strategy

Proportionate interventions evaluated in a randomised controlled trial between 2010 and 2015 inclusive were sought. Electronic searches were undertaken using the databases: MEDLINE (OvidSP), Web of Science (Core Collection) and PsycINFO. The search terms were any of the following in the title or abstract: ‘proportionate universalism’, ‘proportionate intervention’, ‘proportionate treatment’, ‘staged intervention’, ‘staged treatment’, ‘adaptive treatment regimen’, ‘adaptive intervention’, ‘adaptive treatment strategy’, ‘dynamic treatment regimen’, ‘multi-level intervention’ or ‘stepped care’. The search strategy was based on the Cochrane Highly Sensitive Search Strategies for identifying randomised trials [8]. The start date of the 6-year time frame was chosen based on the 2010 publication date of the Marmot review [2], which referred to proportionate universalism. We anticipated no trials would use the term ‘proportionate universalism’ prior to this review and anticipated an increase in the use of such interventions post publication of the Marmot Review [2]. The final search was conducted in March 2016 (after piloting and refining the search strategy). Search strategies were developed that were relevant to the database requirements (see Fig. 1 for the MEDLINE search strategy). The intention of the systematic review was to provide a thorough overview of the types of trials of proportionate interventions being used in practice but not to be exhaustive; therefore, additional hand searching or searching of clinical trial registers was not incorporated.

### Eligibility criteria

All search results that were trials or pilot studies (including protocols) that evaluated interventions delivered proportionate to need were eligible. An intervention proportionate to need was defined as one in which there is a variation in the intervention dependent upon either an intermediate or primary outcome measured prior to the study endpoint. There should be decision stages and at each stage there should be treatment options based on tailoring variables and predefined decision rules. Interventions that are tailored without decision rules were excluded from this review. We excluded observational studies and those not in English. Where more than one article for a single study was found, the main article of published results was included if available and if it superseded any protocol or cost-effectiveness study. We considered all therapeutic areas and imposed no restrictions on the participants.

### Quality control

We did not undertake a quality assessment of the identified studies as the purpose of this review was to understand what interventions and trial designs are being used in practice and how they are being designed and analysed.

### Study selection

Study selection based on the eligibility criteria was performed by review author JCa, who identified relevant results. All duplicates were removed. At the initial screening stage, titles and abstracts were assessed to identify if the study was eligible. The full articles of studies meeting the review criteria were obtained and inspected to identify relevant studies that fulfil the inclusion criteria. Two second authors (JCo and MDT) reviewed a random sample of ten results each to assess agreement and the clarity of eligibility criteria.

### Data extraction and synthesis

A data extraction tool was developed for this review in an Excel spreadsheet. The data extraction tool was piloted by reviewers JCa, JCo and MDT and refined based on feedback. This review evaluated designs and methods used in proportionate intervention trials; therefore, a meta-analysis was not appropriate. We collected the following information with the data extraction tool: publication year, location of study (country), therapeutic area, type of study (trial results, protocol or secondary analysis), design type, aim, eligibility criteria, intervention, tailoring variable, decision rules, number of decision stages, control intervention, final study follow-up period, sample size, primary outcome, overall statistical model, and whether an analysis of different stages was undertaken.

The review results were presented using summary statistics. A narrative synthesis describes any similarities and differences among the included studies. We grouped studies by design type, and study characteristics were tabulated to allow a comparison of the main features.

## Results

### Study selection

**Fig. 2:**
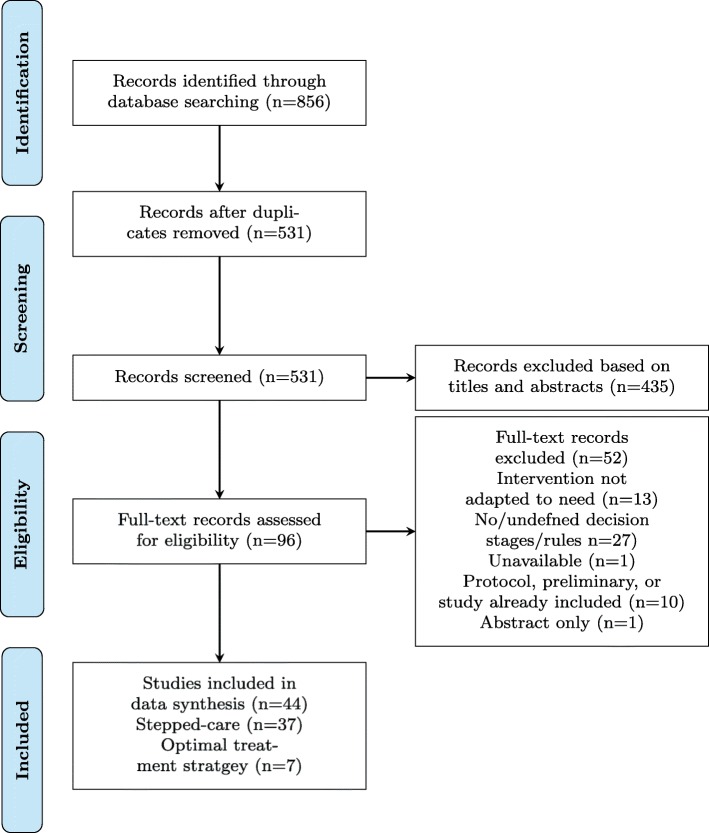
PRISMA study flow diagram. Number of records identified, included and excluded during the literature search

Figure 2 presents the process of study selection in this systematic review. Of the 531 unique records identified from the database search, we identified 44 eligible studies (all papers included are listed in Additional file 2). These 44 studies consisted of 28 trial results, 13 protocols and three secondary analyses. The narrative syntheses have been split into two subcategories by type of study design, stepped care and optimal treatment strategy. Inclusion of a control arm was not required for eligibility. Due to the nature of assessing proportionate interventions, some results did not include a control arm, either due to ethical arguments or because the objective was to identify an optimal treatment strategy.

### Study characteristics

Table 1 presents an overview of the included studies. There were 18 studies based in the United States, 14 in the Netherlands, one in the Netherlands and Belgium, two in each of Australia, England and Scotland, Norway and Sweden, and three based in other countries (India, Nigeria and a multi-site study in France, Hungary, Romania and Slovakia).

**Table 1:** Overview of studies included in the systematic review

First author	Date ^*a*^	Therapeutic area	Country	Follow-up ^*b*^	*N* ^*c*^
Ell [54]	2010	Depression and anxiety	United States	12	387
Van’t Veer-Tazelaar [55]	2010	Depression and anxiety	The Netherlands	12	170
Braamse [56]	2010	Distress after autologous stem cell transplantation	The Netherlands	10	286
Patel [57]	2010	Depression and anxiety	India	12	2796
Gilliam [25]	2010	Obsessive–compulsive disorder	United States	3	14
Kay-Lambkin [58]	2010	Depression among methamphetamine users	Australia	5	8
Richter [18]	2011	Blood pressure	France, Hungary, Romania, Slovakia	6	256
Weiss ^*d*^ [32]	2011	Prescription opioid dependence	United States	6	653
Mitchell [16]	2011	Bulimia nervosa	United States	12	293
Seekles [59]	2011	Depression and anxiety	The Netherlands	6	120
Tolin [23]	2011	Obsessive–compulsive disorder	United States	3	34
van der Leeden [26]	2011	Anxiety in children	The Netherlands	6	133
Apil [60]	2012	Depression	The Netherlands	12	136
Karp [61]	2012	Depression and chronic pain	United States	12	250
Shortreed ^*d*^ [33]	2012	Schizophrenia	United States	18	1460
Dozeman [62]	2012	Depression and anxiety	The Netherlands	10	185
Nordin [24]	2012	Stress management of cancer patients	Sweden	12	300
Jakicic [11]	2012	Weight loss	United States	18	363
Wang ^*d*^ [34]	2012	Oncology	United States	7	150
Pommer [63]	2012	Depression and anxiety in patients with asthma or COPD	The Netherlands	24	160
Lamb [17]	2012	Whiplash injuries	England and Scotland	12	3851
Krebber [27]	2012	Distress in head and neck and lung cancer patients	The Netherlands	12	176
Borsari [21]	2012	Alcohol consumption	United States	9	598
Rose ^*d*^ [30]	2013	Smoking cessation	United States	6	606
Watson [13]	2013	Alcohol consumption	England and Scotland	12	529
Oosterbaan [29]	2013	Common mental disorders	The Netherlands	8	163
van Dijk [64]	2013	Depression among patients with diabetes and/or coronary heart disease	The Netherlands	12	236
Arving [65]	2013	Stress management of cancer patients	Norway	24	300
Mattsson [28]	2013	Depression and anxiety	Sweden	24	200
Carels [12]	2013	Weight loss	United States	4	52
van der Aa [66]	2013	Depression and anxiety	The Netherlands and Belgium	24	230
Kasari ^*d*^ [31]	2014	Communication for minimally verbal children with autism	United States	8	61
Muntingh [67]	2014	Panic and anxiety	The Netherlands	12	180
Kilbourne ^*d*^ [36]	2014	Mood disorder	United States	24	1600
Hamall [19]	2014	Families living with childhood chronic illness	Australia	6	1050
Gureje [68]	2015	Depression	Nigeria	12	1190
Stoop [69]	2015	Depression and anxiety in patients with diabetes, asthma or COPD	The Netherlands	18	46
Stam [20]	2015	Impairment in older dizzy people	The Netherlands	12	300
Lock [15]	2015	Anorexia nervosa	United States	6	45
Schuurhuizen [70]	2015	Distress in patients with metastatic colorectal cancer	The Netherlands	11	715
Haug [71]	2015	Panic and anxiety	Norway	12	173
Salloum [72]	2015	Post-traumatic stress in children	United States	3	53
Wu ^*d*^ [35]	2015	Bipolar disorder	United States	3	365
Painter [73]	2015	Depression in HIV patients	United States	12	249

*COPD* chronic obstructive pulmonary disease

^a^Publication date

^b^Final follow-up post baseline in months

^c^Sample size

^d^Optimal-treatment strategy subcategory

The median number of decision stages (points at which the intervention was adapted according to need based on predefined decision rules) was 2 (interquartile range 1 to 3). The median length of trial follow-up was 12 months (interquartile range 6 to 12 months) and the median sample size was 236 (interquartile range 150 to 387).

### Stepped care

Table 2 is a summary of the included stepped-care studies. A total of 84% (37 of 44) of the studies followed a stepped-care model for the intervention. The stepped-care model is recommended by the National Institute for Health and Care Excellence (NICE) [9] for the provision of services for common mental health disorders. In a stepped-care model, the least intensive intervention (or the lowest level of intervention) is delivered first to all patients, and patients step up or down the stepped-care treatment pathway dependent upon their response to the previous intervention step.

**Table 2:** Characteristics of included stepped-care studies

First author	Intervention	Tailoring variable and decision rules (response unless otherwise stated)	Primary outcome	Statistical analysis	Analysis of stages
Ell [54]	Stepped care, three steps: (1) based on patient preference, patients start PST or antidepressant medication, 8 weeks, (2) a different antidepressant medication or the addition of antidepressant medication or PST, 4 weeks, (3) considered for additional PST, augmentation of low-dose trazodone for insomnia and referral to speciality mental health care	50% SCL-20 reduction	Depression remission was assessed by SCL-20 < 0.5 or PHQ-9 < 5	Logistic regression model used to compare the odds of achieving clinically meaningful improvement between treatment groups	No
Van’t Veer-Tazelaar [55]	Stepped care, four steps: (1) watchful waiting, (2) bibliotherapy, (3) PST, (4) antidepressant medication; stages were in 3-month cycles	CES-D < 16	MINI/DSM-IV diagnostic status of depressive and anxiety disorders	Incremental effectiveness computed as the difference in the probability of a disorder-free period between groups	No
Braamse [56]	Stepped care, two steps: (1) internet-based self-help programme, (2) contracting, individual face-to-face counselling, medication or referral to other services	PHQ-9 ≤ 10 and/or HADS < 8 and/or STAI < 40	Psychological distress using HADS and physical role function using EORTC-QLQ-C30	ANOVA	No
Patel [57]	Stepped care, four steps: (1) psychoeducation, (2) antidepressants, (3) interpersonal psychotherapy in addition to antidepressants or an alternative to antidepressants for those who did not respond to them, (4) referral to psychiatrist	Varying	ICD-10 diagnosis	Chi-squared and *t*-tests; mixed-effects models for longitudinal data	No
Gilliam [25]	Stepped care, two steps: (1) short therapist sessions and bibliotherapy, (2) longer therapist-directed sessions	Y-BOCS reduction ≥5 points plus a post-treatment score of ≤13	Y-BOCS total score and the clinician’s CGI severity rating	Repeated measures ANOVA	No
Kay-Lambkin [58]	Stepped care, four steps: (1) brief integrated CBT/MI intervention, one session, (2) four CBT/MI sessions, (3) four CBT/MI sessions, (4) four CBT/MI sessions	Varying	Depression and methamphetamine use	Small sample size, so no statistical analyses	No
Richter [18]	Stepped care, six steps: incremental therapy included the following add-on therapies at 4-week intervals: aliskiren 150–300 mg once daily, hydrochlorothiazide 12.5–25 mg once daily and finally amlodipine 5–10 mg once daily, as needed	Meet the target blood pressure at 4-week intervals	Estimated cumulative probability of patients achieving blood pressure target	Probability of reaching the blood pressure target, assessed by estimating control rates of patients who reached target per visit using life-table survivor estimates at each visit; summaries presented of change in blood pressure per treatment step	Yes
Mitchell [16]	Stepped care, three steps: (1) therapist-assisted self-help for 18 weeks, (2) fluoxetine until 1-year follow-up, (3) full CBT for 6 months	70% or more reduction in frequency of purging by the end of Session 6	Recovery (no binge eating or purging behaviours in the past 28 days); remission (no longer meeting DSM-IV criteria)	ANOVA with the site × treatment interaction	No
Seekles [59]	Stepped care, four steps: (1) watchful waiting, 4 weeks, (2) guided self-help, (3) five short face-to-face PST sessions, (4) pharmacotherapy and/or specialised mental health care	IDS < 14 and HADS < 8 and WSAS < 6	IDS and HADS	*t*-tests to compare scores between two groups	No
Tolin [23]	Stepped care, two steps: (1) bibliotherapy, 6 weeks, (2) therapist-directed ERP sessions	Y-BOCS ≥5 and ≤13	Y-BOCS and cost	Mixed-effects model	No
van der Leeden [26]	Stepped care, four steps: (1) randomised to group or individual CBT sessions for children and parents, (2) five manual-based PCTA sessions, (3) additional five PCTA sessions	Children diagnosed with an anxiety disorder or who scored below the cut-off of the MASC	Change in proportion of children with any DSM-IV anxiety disorder	Percentages of children free of any anxiety disorder after each treatment phase and by intervention, e.g. intervention 1 only, 1 and 2, 1–3 and all combined; mixed-effects models for changes on the continuous variables	Yes
Apil [60]	Stepped care, four steps: (1) watchful waiting, 6 weeks, (2) bibliotherapy self-help booklet, 6 weeks, (3) 12 individual CBT weekly sessions, (4) referral to physician or psychotherapist for any indicated treatment	CES-D ≤16	Incidence of new depressive episode	Feasibility evaluated descriptively; chi-squared test used to test if selective drop-out biased results of incidence of a new depressive episode	No
Karp [61]	Stepped care, two steps: (1) 6 weeks open treatment with venlafaxine xr 150 mg/day and supportive management, (2) 14 weeks in which non-responders are randomised to high-dose venlafaxine xr (up to 300 mg/day) with PST for depression and pain or high-dose venlafaxine xr and continued supportive management	PHQ-9 of ≤5 for 2 weeks and at least 30% improvement in the average numeric rating scale for pain	Univariate pain and depression response and both observed and self-report disability	Number needed to treat between two interventions; repeated measures mixed-effect models for self-reported and observed physical disability between the two interventions across time	No
Dozeman [62]	Stepped care, four steps: (1) watchful waiting, 3 months, (2) activity scheduling, 3 months, (3) life review and consultation with GP, 3 months, (4) consultation with GP to discuss further treatment, 3 months	Improvement of ≥5 points on CES-D	Incidence of major depressive disorder or anxiety disorder using MINI	Incidence rate ratio using an unadjusted and adjusted Poisson regression analysis of MINI/DSM-IV depressive and anxiety cumulative incidence (1 = developed a disorder and 0 = remained disorder-free) on the treatment indicator	No
Nordin [24]	Stepped care, two steps: (1) low-intensity stress-management intervention given to all patients, (2a) more intensive group stress management treatment, (2b) more intensive individual stress management treatment	Decrease in stress-related symptoms measured by IES or HADS from clinical levels to normal results	Subjective distress (intrusion and avoidance) assessed by IES	Repeated measures ANOVA (continuous variables) and chi-squared test (categorical variables)	No
Jakicic [11]	Stepped care, six steps: (1) monthly group intervention session plus weekly mailed lessons and submission of self-monitoring diaries, (2) continue step 1 plus 10-minute monthly telephone contact, (3) step 2 plus second 10-minute telephone contact each month, (4) step 3 plus 1 individual in-person intervention contact per month, (5) step 4 plus meal replacement shakes and bars provided to replace one meal and one snack per day, (6) step 5 plus replace one telephone contact with second individual session per month; modified based on weight-loss achievement at 3-month intervals	Weight-loss goals 5% at 3 months, 7% at 6 months, 10% at 9 months, and remained at 10% at 12, 15 and 18 months	Change in weight over 18 months	*t*-test to compare mean weight loss between groups; mixed-effects models for longitudinal data	No
Pommer [63]	Stepped care, three steps: (1) four sessions of extensive psycho-education, (2) a course on coping with depression and/or anxiety, 10 consultations, (3) coaching (six booster sessions on top of step 2) complemented with optional antidepressant and/or anxiolytic medication	PHQ-9 < 7 and/or GAD-7 < 8	PHQ-9, GAD-7 and MINI	Chi-squared and *t*-tests; mixed-effects models for longitudinal data	No
Lamb [17]	Stepped care, two steps: (1) Whiplash Book advice or active management advice, (2a) single session of physiotherapist advice or (2b) up to six sessions of physiotherapy	Non-response if persistent symptoms 3 weeks after emergency department attendance (WAD grades I–III)	Neck Disability Index	Mixed models to account for clustering effects from NHS trusts and therapists in step 2	Yes
Krebber [27]	Stepped care, four steps: (1) watchful waiting, 2 weeks, (2) guided self-help via internet or booklet, 5 weeks, plus six phone or email coaching sessions, (3) PST administered by a specialised nurse, (4) specialised psychological intervention or antidepressant medication chosen in cooperation between patient and care co-ordinator	HADS-A or HADS-D ≤ 7	HADS	Repeated measures ANOVA (continuous outcomes); generalised estimating equations used to evaluate longitudinal changes	No
Borsari [21]	Stepped care, two steps: (1) brief advice session, (2a) brief motivational intervention, (2b) assessment only	Non-response if student has heavy episodic drinking ≥4 and/or alcohol-related consequences ≥5 in the past month they were randomised to receive step 2 or control (assessment only)	Heavy episodic drinking and peak blood alcohol content	Comparison of outcomes at 3, 6 and 9 months between those assigned to (2a) or (2b) using generalised estimating equations for longitudinal data	Yes
Watson [13]	Stepped care, three steps: (1) behavioural change counselling, one session, (2) motivational enhancement therapy, three sessions, (3) local specialist alcohol services	Three-item AUDIT-C <5	Average drinks per day	Linear mixed model, to account for variation in GP practice and allocated therapist	No
Oosterbaan [29]	Stepped care, two steps: (1) self-help course, (2) CBT in combination with antidepressant medication	CGI-S < 3	% of patients responding to and remitting after treatment measured using CGI-S	Logistic mixed-effects models; analysis after steps 1 and 2	Yes
van Dijk [64]	Stepped care, four steps: (1) watchful waiting, (2) guided self-help, (3) PST, (4) referral to GP	PHQ-9 ≥ 6	Cumulative incidence of DSM-IV major depressive disorder using MINI	Logistic mixed-effects models	No
Arving [65]	Stepped care, two steps: (1) low-intensity stress management consisting of two counselling sessions over 6 weeks, (2) more intensive stress-management treatment consisting of 4–7 sessions	IES and HADS score at 6-week assessment not clinically significant	Avoidance and intrusions	Repeated measures ANOVA (continuous variables) and chi-squared test (categorical variables)	No
Mattsson [28]	Stepped care, two steps: (1) self-help material, chat forum and FAQ section, (2) CBT	HADS subscale <7 at 1, 4 or 7 months after inclusion	HADS, 20% change as clinically relevant	Repeated measures ANOVA to compare intervention and control group regarding anxiety, depression, post-traumatic stress and health-related QoL	No
Carels [12]	Stepped care, three steps: (1) group-based behavioural weight-loss programme, 6 weeks, (2a) behavioural weight-loss programme, 6 weeks or (2b) self-help, (3a) behavioural weight-loss programme, 6 weeks or (3b) self-help	Meet the 3% weight-loss target	% weight loss	Repeated measures ANOVA (continuous variables) and chi-squared test (categorical variables) to compare differences between treatment groups at the end of each stage and the end of the whole intervention	Yes
van der Aa [66]	Stepped care, four steps: (1) watchful waiting, (2) guided self-help, (3) PST, (4) referral to GP	CES-D < 16 or HADS-A < 7	MINI	Survival analysis and mixed-effects model	No
Muntingh [67]	Stepped care, four steps: (1) guided self-help, (2) CBT, six sessions, (3) antidepressant medication prescribed by GP, (4) optimisation of medication in primary care or referral to secondary care	50% reduction in BAI score and BAI ≤ 11	BAI score	Difference in gain BAI gain scores from baseline; inverse probability weighting used, accounts for variation in receiving treatment	No
Hamall [19]	Stepped care, three steps: (1) family resilience and well-being fact sheet, (2) family resilience and well-being activity booklet, (3) family resilience information support group or waiting list control	Step 2: parents eligible if have a child attending one of four selected outpatient clinics at the paediatric hospital. Step 3: eligible if K10 ≥ 15	Parental well-being (K10); family functioning (McMasters Family Assessment Device); social connectedness (Medical Outcomes Study Social Support Survey); family beliefs	Descriptive statistics used for step 1. ANOVA for effect of booklet intervention for all participants in step 2 and sustained change tested using a repeated measures mixed-effects model for the participants who did not move into step 3. ANOVA to examine additional effect of the information support group relative to waiting list control group	Yes
Gureje [68]	Stepped care, three steps: (1a) eight weekly psychoeducation and PST sessions, (1b) eight weekly psychoeducation and PST sessions plus doctor’s advice on treatment, (2a) four monthly psychoeducation and weekly PST sessions, (2b) eight weekly psychoeducation and PST sessions, (2c) consult doctor plus eight weekly psychoeducation and PST sessions, (3a) four monthly psychoeducation and weekly PST sessions, (3b) consult doctor plus eight weekly psychoeducation and PST sessions	Step 1: (1a) if PHQ-9 = 11–14, (1b) if PHQ-9 ≥ 18. Step 2: (2a) PHQ-9 < 11, (2b) PHQ-9 = 11–17, (2c) PHQ-9 ≥ 18. Step 3: (3a) PHQ-9 < 11, (3b) PHQ-9 ≥ 11	Recovery from depression at 12 months as shown by PHQ-9 ≤ 6	Mixed-effects regression model	No
Stoop [69]	Stepped care, three steps: (1) four weekly psychoeducation individual meetings, (2) 10 weekly individual meetings covering the coping with depression/anxiety course, (3) advice to meet GP to discuss optional medication and six booster sessions during 6 months; followed by monitoring of symptoms of depression or anxiety if remission	PHQ-9 < 7 and/or GAD-7 < 8	Symptoms of anxiety and depression after 12 months of intervention and 6 months post-intervention	ANCOVA and clinical significance in terms of effect size	No
Stam [20]	Risk-factor-guided intervention including: (1) medication adjustment if three or more prescribed fall-risk-increasing drugs, (2) stepped care if anxiety disorder or depression, (3) exercise therapy if impaired functional mobility; those eligible for more than one intervention start them at the same time. Stepped care, four steps: (1) watchful waiting, 6 weeks, (2) guided self-help treatment, 6 weeks, (3) PST maximum six sessions, (4) referral to GP	GAD-7 < 10, PHQ-9 < 10, or positive PHQ-PD score	Dizziness-related impairment, assessed using the Dizziness Handicap Inventory	Mixed-effects models for longitudinal data to compare intervention and control groups, regardless of number of interventions; separate subgroup analyses for three groups that received one of three interventions	No
Lock [15]	Adaptive intervention, intensive family coaching, consisting of FBT/IPC: four sessions of FBT plus three sessions of IPC	Weight gain ≥2.3 kg after FBT, proceed to IPC	Retentions and treatment use, suitability and expectancy, clinical outcomes, changes in parental self-efficacy	Feasibility and acceptability compared across the randomised groups (FBT versus FBT/IPC) using chi-squared test and *t*-test	No
Schuurhuizen [70]	Targeted selection by a nurse (HADS ≥ 13 or Lastmeter ≥ 5), enhanced care (treatment managed by a trained nurse) and stepped care. Stepped care, four steps: (1) watchful waiting, 3 weeks, (2) guided self-help programme, 5–7 weeks, maximum six sessions in 10 weeks, (3) face-to-face PST, (4) psychotherapy, medication or a referral to other services (e.g. social work)	HADS < 13	Psychological distress measured by HADS	ANCOVA for difference between groups; time patients entered stepped care and the response to treatment (progression or not) are accounted for via a covariate	No
Haug [71]	Stepped care, three steps: (1) short psychoeducation, (2) 10 weeks’ internet-based self-help programme, (3) 12 weeks of individual CBT	Two out of three of the following criteria: (1) loss of primary diagnosis (SCID-I), (2) CSR ≤ 3 and reduced by at least two points, (3) for panic disorder, BSQ ≤ 2.5, and for seasonal affective disorder, SPS ≤ 25	CSR, a 0–8 severity rating of the primary anxiety diagnosis	Multiple regression analyses enhanced with the full information; maximum likelihood estimation of missing data	No
Salloum [72]	Stepped care, two steps: (1) three therapist-led sessions, 11 parent–child meetings at home over 6 weeks using a workbook, weekly brief phone support, online psychoeducation information and video demonstrations, (2) nine trauma-focussed CBT sessions	PTS ≤ 3, or TSCYC-PTS ≤ 39 and an CGI-I rating of 3, 2 or 1	TSCYC-PTS	Linear mixed-effects models (continuous outcomes); generalised linear mixed-effects models (non-continuous outcome) for longitudinal data	No
Painter [73]	Stepped care, five steps: (1) watchful waiting, (2) depression care team treatment suggestions (counselling or pharmacotherapy, considering participant preference), (3) pharmacotherapy suggestions after review of treatment history, (4) combination pharmacotherapy and speciality mental health counselling, (5) referral to speciality mental health	Non-response defined on five different measures: antidepressant adherence, counselling non-adherence, report of severe adverse effects, increase in PHQ-9 from baseline by ≥5 or <50% decrease from enrolment PHQ-9	Quality-adjusted life years and percentage of participants with depression treatment response	Generalised linear models to calculate predicted expenditure for each participant to determine incremental cost; logistic regression models to compare the odds of achieving clinically meaningful improvement (SCL-20 improved by ≥50%) between groups	No

*ANCOVA* Analysis of covariance; *ANOVA* analysis of variance; *AUDIT-C* Alcohol Use Disorders Identification Test, Consumption; *BAI* Beck Anxiety Inventory; *BSQ* Body Sensations Questionnaire; *CBT* cognitive behavioural therapy; *CES-D* Epidemiologic Studies Depression Scale; *CGI* Clinical Global Impression; *CGI-I* Clinical Global Impression, Improvement Scale; *CGI-S* Clinical Global Impression, Severity Scale; *CSR* Clinicians’ Severity Rating; *DSM-IV* Diagnostic and Statistical Manual of Mental Disorders; *EORTC-QLQ-C30* European Organisation for Research and Treatment of Cancer Quality of Life Questionnaire; *FAQ* Frequently asked questions; *FBT* Family-based Treatment; *GAD-7* Generalised Anxiety Disorder, 7; *GP* general practitioner; *HADS* Hospital Anxiety and Depression Scale; *HADS-A* Hospital Anxiety and Depression Scale, Anxiety; *HADS-D* Hospital Anxiety and Depression Scale, Depression; *ICD-10* International Statistical Classification of Diseases and Related Health Problems, 10th revision; *IDS* Inventory of Depressive Symptomatology; *IE*; *IES* Impact of Events Scale; *IPC* Intensive Parental Coaching; *K10* Kessler Psychological Distress Scale; *MASC* Multidimensional Anxiety Scale for Children; *MI* motivational interview; *MINI* Mini International Neuropsychiatric Interview; *NHS* National Health Service; *PCTA* Parent–Child Treatment for Anxiety; *PHQ-9* Patient Health Questionnaire; *PHQ-PD* Patient Health Questionnaire, Panic Disorder Subscale; *PST* problem-solving treatment; *QoL* quality of life; *SCID-I* Structured Clinical Interview for DSM-IV; *SCL-20* 20-item Symptom Checklist Depression Scale; *SPS* Sensory Processing Sensitivity; *STAI* State-Trait Anxiety Inventory; *TSCYC-PTS* Trauma Symptom Checklist for Young Children, Post-Traumatic Stress Subscale; *WAD* Whiplash-Associated Disorders; *WSAS* Work and Social Adjustment Scale; *Y-BOCS* Yale–Brown Obsessive–Compulsive Scale

**Fig. 3:**
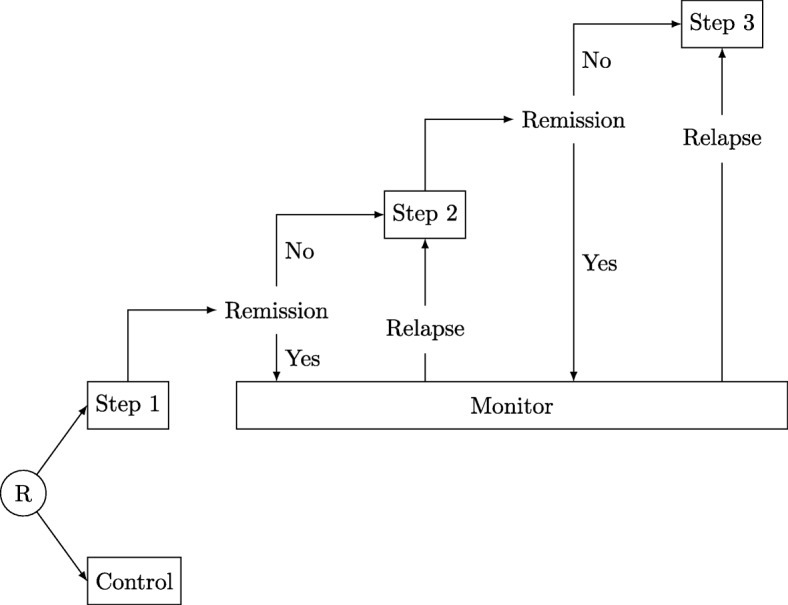
Example of a stepped-care trial with three steps and the option to rejoin treatment if relapse occurs. R randomise

Figure 3 represents the flow of patients through an example of a typical stepped-care trial with three treatment steps. The key principles of stepped care are to provide the most appropriate and best treatment according to need, to reduce the burden on patients by providing only the treatment required and to improve cost-effectiveness by providing the level of intervention required for a positive outcome [9]. The reduction of costs for those who respond to lower-intensity interventions can free up resources for those who require more intensive treatment [10].

The majority of stepped-care studies (73%; 27 of 37) focussed on the therapeutic areas of depression, anxiety, stress or some form of mental health disorder. Other therapeutic areas targeted included: weight loss [11, 12], alcohol consumption [13, 14], eating disorders [15, 16], whiplash injuries [17], blood pressure control [18], resilience and well-being of families living with childhood chronic illness [19], and impairment in older dizzy people [20].

The interventions often involved some form of watchful waiting period for the first step followed by regular monitoring at predefined follow-up times of an outcome measure (either secondary or primary). Based on this outcome measure, decisions were made whether to progress to the next step or not. This process continued for however many steps were included in the intervention. Based on the individual’s outcome, at each decision stage the options for the following step were often: (1) a choice of treatments, (2) continue with the same treatment, (3) augment the treatment or (4) discontinue the treatment altogether. The progression of treatment steps for interventions aimed at mental health disorders commonly included a watchful waiting period, bibliotherapy, guided self-help, or psychotherapy sessions (either individual or group based), with a possible progression to medication (for example, antidepressants).

Control conditions were generally usual care or enhanced usual care with others being assessment only [19], waiting list control [21] or the active treatment delivered in a non-stepped model [12, 22, 23]. Four of the stepped-care trials had no explicit control [18, 24–26]. In one study, it was argued that the lack of control was partially inherent to the stepped-care design since it was unethical to assign individuals to a waiting list control after the first treatment step if they needed further treatment [26].

Measures were taken at baseline, at each decision stage and after the end of the final intervention stage. Generally, follow-up measures were also taken a number of months after completion of the interventions to assess if the effects were sustained. Our search results included both individually randomised and cluster-randomised trials.

A variety of statistical analysis methods were used, dependent upon the outcome measures and main aims. Longitudinal data were incorporated into many of the analyses. Mixed-effects models, containing both fixed and random effects, were used as the statistical analysis method in 38% of studies (14 of 37; Table 2). They were used to account for both longitudinal data and the clustering effects of National Health Service (NHS) trusts, therapists and other health professionals. Repeated measures analysis of variance (ANOVA) was used in three studies [25, 27, 28]; however, this method does not successfully deal with missing values. In contrast, mixed-effects models assume data are missing at random and they allow for imbalanced or missing observations within patient.

Six stepped-care studies included or planned some form of analysis of the different stages. These included:

1. summaries of outcome measures presented per treatment step [18],
2. an analysis of outcomes after steps 1 and 2 [29],
3. an analysis at the end of each step and the end of the whole intervention as well as a comparison of differences in weight loss and self-monitoring characteristics between those who were stepped down and those who continued to receive treatment in the stepped-care arm [12],
4. a planned analysis of demographic data to compare the characteristics of those who agreed to participate in steps 2 and 3 compared to those who declined (for eligible patients) [19],
5. the percentages of children free of any anxiety disorder after each treatment phase and by intervention [26],
6. analysis of outcomes after step 1 and analysis of outcomes after step 2 adjusting for the intervention received in step 1 and any interactions between the step 1 and step 2 interventions [17].

The objectives of Lamb et al. [17] were to evaluate the effectiveness of step 1, step 2, and the combined effects of the treatments together. This was made possible by designing two linked pragmatic randomised controlled trials. In step 1, emergency departments were cluster randomised to the Whiplash Book or usual care, and individual consent was not sought at this stage. In step 2, participants who received either of the step 1 treatments and were eligible after step 1 (persistent symptoms at 3 weeks) were individually randomised at step 2 to either one physiotherapist advice session or up to six physiotherapist advice sessions.

### Optimal adaptive treatment strategy

Table 3 presents a summary of the studies of optimal adaptive treatment strategies. A total of 16% (7 of 44) of the review studies were aimed at finding an optimal treatment strategy. Treatments consist of more than one phase. Unlike most of the stepped-care studies, randomisation occurs more than once and there was often no true control, since the different adaptive treatment strategies were compared to one another. Six of the studies were explicitly defined as SMARTs, with the other study based on a two-phase trial design evaluating an adaptive smoking cessation treatment strategy [30].

**Table 3:** Characteristics of included optimal treatment strategy studies

First author	Intervention	Tailoring variable and decision rules (response unless otherwise stated)	Primary outcome	Statistical analysis	Analysis of stages
Weiss [32]	Two-stage intervention. Stage 1: buprenorphine–naloxone induction, 2 weeks of stabilisation, a 2-week taper and 8 weeks of follow-up. Stage 2: 12 weeks of buprenorphine–naloxone stabilisation, a 4-week taper and 8 weeks of follow-up. In each phase, patients were randomised to (1) standard medical management or (2) standard medical management plus individual drug counselling	Stage 1: self-reported opioid use on ≤4 days in a month, absence of two consecutive opioid-positive urine test results, no additional substance use disorder treatment and ≤1 missing urine sample. Stage 2: abstaining from opioids during week 12 and during ≥2 of the previous 3 weeks	Composite measures indicating minimal or no opioid use based on urine test-confirmed self-reports	Compare two treatment conditions using the stage 2 endpoint; generalised estimating equations to account for clustering of patients by site	Yes
Shortreed [33]	Two-stage intervention. Initially randomised to newer atypical antipsychotics or to perphenazine. Patients randomised at stage 1 to perphenazine who discontinue were randomised to a newer atypical antipsychotic. Patients randomised at stage 1 to a newer atypical antipsychotic who discontinue were given the choice of two randomisation arms: (1) with ziprasidone, olanzapine, risperidone or quetiapine, excluding their previous treatment or (2) with clozapine, olanzapine, risperidone or quetiapine, again excluding their previous treatment. Dissatisfied patients could opt to switch treatment again; at this stage treatment was neither randomised nor blinded	Non-response if patient discontinues treatment and then eligible for randomisation to next stage	12-month PANSS score and 12-month QoL score	Marginal structural modelling using a weighted analysis to compare treatment regimes: the always atypical antipsychotic regime or the perphenazine and atypical regime	No
Wang [34]	Three-stage intervention. Stage 1: randomised to one of four combination chemotherapies. Stage 2: (2a) responders receive second course of same chemo, (2b) non-responders randomised to second-line treatment. Stage 3: After (2a): (3a) responders receive second course of same treatment, (3b) if treatment not finished. After (2b): (3c) if overall success, finish treatment, (3b) if not randomised to second treatment, process repeated once more. After (3a): finish treatment	Response defined as: prostate-specific antigen (PSA) decline of at least 40% from baseline, objective regression (of any magnitude) of any measurable disease, improvement in any cancer-related symptom and no new lesions or new cancer-related symptoms. Success defined as PSA decline of at least 80% from baseline, resolution of all cancer-related symptoms, an objective tumour regression of at least 50% from baseline for all measurable lesions and no new lesions or cancer-related symptoms	Long-term survival using log survival time. Efficiency in diminishing disease burden over 32 weeks using three specific scoring functions defined as functions of toxicity and efficacy taking values in the interval [0,1]	Inverse probability weighting methods to estimate the mean of counterfactual outcome for dynamic treatment regimens and sequentially randomised trials	No
Rose [30]	Two-stage intervention. Stage 1: all received nicotine patch treatment 2 weeks before quit date. Responders continue nicotine patch treatment. Non-responders randomised to (1) control (nicotine patch), (2) nicotine patch and bupropion or (3) varenicline alone. Stage 2: for pre-cessation nicotine patch responders, nonlapsers continue nicotine patch and for those who lapsed in the first week after quit date randomised to (1) control (nicotine patch), (2) nicotine patch and bupropion or (3) varenicline alone	Ad lib smoking (expired carbon monoxide levels) decreased by >50% after 1 week	Continued smoking abstinence at end of treatment	Logistic regression compared each rescue treatment against the control	Yes
Kasari [31]	Two-stage intervention. Stage 1: sessions of (a) JASP+EMT or (b) JASP+EMT+SGD. Stage 2: early responders continue stage 1 treatment. Slow responders from (1a) randomised to receive intensified stage 1 treatment or augmented stage 1. Slow responders from (1b) receive intensified stage 1 treatment	After stage 1, if child demonstrated 25% or greater change on at least half of the variables (7 out of 14), then the participant was considered an early responder	Total spontaneous, communicative utterances coded from a standardised Natural Language Sample	Mixed-effects models compared outcome between stage 1 treatments. Secondary aim analysis used a weighted regression to compare mean outcomes between the three embedded adaptive interventions, including an indicator for stage 1 and 2 treatments	Yes
Kilbourne [36]	SMART design for adaptive implementation strategy. Run-in phase: sites offered REP to implement life goals for patients with mood disorders. Sites not initially responding to REP are randomised to receive additional support from an EF or both EF/IF. Additionally, sites randomised to EF and still not responsive will be randomised to continue with EF alone or to receive EF/IF	<50% patients receiving ≥3 evidence-based practice sessions	SF-12 mental-health-related QoL and PHQ-9 scores	Linear mixed-effects models. Compare interventions in non-responding sites beginning with REP plus EF/IF versus interventions beginning with REP plus EF on longitudinal patient-level change in number of life-goal sessions received. Compare whether continuing REP plus EF versus augmenting with REP plus EF/IF leads to changes in outcomes, among sites who are non-responsive to REP plus EF at month 12	Yes
Wu [35]	Two-stage intervention. Stage 1: patients randomised to bupropion, paroxetine or placebo. Stage 2: non-responders assigned second intervention. If receiving bupropion or paroxetine at stage 1, current doses increased. If placebo at stage 1, bupropion or paroxetine	≥50% improvement over initial SUMD and not meeting DSM-IV criteria for hypomania or mania	SUMD	Q-learning to estimate optimal regime	Yes

*DSM-IV* Diagnostic and Statistical Manual of Mental Disorders; *EF* external facilitator; *EMT* Enhanced milieu teaching; *IF* internal facilitator; *JASP* Joint Attention Symbolic Play Engagement and Regulation; *PANSS* Positive and Negative Syndrome Scale; *PHQ-9* Patient Health Questionnaire; *PSA* prostate-specific antigen; *QoL* quality of life; *REP* Replicating Effective Programmes; *SF-12* 12-Item Short Form Health Survey; *SGD* speech-generating device; *SMART* Sequential Multiple Assignment Randomised Trial; *SUMD* Scale to Assess Unawareness of Mental Disorder

The optimal treatment strategy studies included three with trial results [30–32], three secondary analyses of trials [33–35] and one trial protocol [36]. All seven studies were based in the United States. Therapeutic areas covered were oncology [34], schizophrenia [33], depression and anxiety [36], bipolar disorder [35], patients dependent on prescription opioids [32], smoking cessation [30] and communication for minimally verbal children [31].

**Fig. 4:**
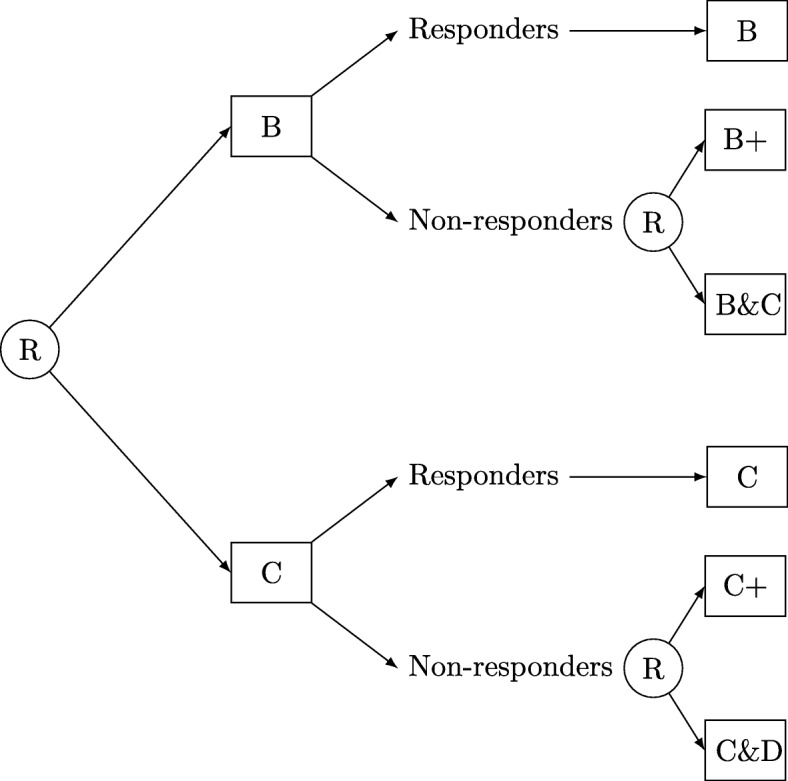
SMART design with second randomisation dependent upon an intermediate outcome response status. SMART Sequential Multiple Assignment Randomised Trial, R randomise, B/B+, C/C+ and D represent different treatments, with for example, B+ being the more intense version of B

Five of the optimal treatment strategy studies were based on two phases or stages of intervention and two studies used a three-phase design [34, 36]. A measurement at the end of each phase was used to assess the response and thus, progression to the next phase. Participants were generally randomised to phase 1 treatments. If they were classified as responders to phase 1, they continued this treatment whereas non-responders were randomised to the following phase treatments. An example design is represented in Fig. 4. The number of treatments at each randomisation phase varied greatly between studies. In phase 1, there were between two and six treatments randomised (two treatments [31, 32, 36], three treatments [35], four treatments [34] or five treatments [33]). No control group was used in four of the studies [31, 33, 34, 36]. One study used a placebo in stage 1 [35] and usual care was used in another [32].

In general, more complex analysis methods were used for the optimal adaptive treatment strategies compared to the stepped-care trials. Inverse probability weighting methods were used to estimate the outcome means associated with each of the two-stage dynamic treatment regimes [34]. A comparison of two treatment conditions was done using the phase 2 endpoint and generalised estimating equations (to account for correlation among measurements of patients from the same site) [32]. Other methods to estimate the optimal treatment strategy included: Q-learning [35], marginal structural models [33] and mixed-effects models [31, 36]. The studies were generally interested in estimating the optimal treatment strategy as a whole rather than considering the effects of each treatment stage. Different stages of the interventions were considered in some analyses, including measuring those who responded after stage 1 and randomising those who did not respond to stage 2 [32] and weighted regression to compare outcomes between the three embedded adaptive treatments, including an indicator for stage 1 and stage 2 treatments and accounting for the probability of a participant following their assigned sequence of treatments based on randomisation sequence [31].

## Discussion

### Main findings

The results suggest that trials are being designed in various therapeutic areas that fit the proportionate universal framework. Most results were conducted in developed countries. The term ‘proportionate universalism’ was not used within the identified studies. Other terminology used included: stepped care, adaptive treatment strategy, dynamic treatment regimen and SMART. In the review, eligible studies fell into two main subcategories of designs: trials using the stepped-care design (to provide treatment dependent on need) or aimed at identifying an optimal treatment strategy (when more than one treatment was available at various stages and administered dependent upon need). The stepped-care model begins with a lower level of intervention at the first step and treatment is administered at further steps only to those in need. Randomisation generally occurs only at baseline. The optimal treatment strategy trials inform decisions on how and when to alter treatment. They generally involved randomisation at each stage dependent upon the response at the end of the previous stage.

Mental health disorders were the most common therapeutic area of research in this review. This is most likely because a large majority of the results were stepped-care trials, which is a NICE-recommended pathway for mental health care [9]. Reasons for using a proportionate intervention were mainly based around costs and providing the level of care required by an individual. This is particularly relevant in mental health and complex interventions, since they are often fairly resource-intensive (both in time and costs).

The statistical methods used varied greatly based on the outcome measures, though longitudinal data are generally a feature of trials of a proportionate intervention. The trials need to update and measure the adjusting needs of patients during delivery of the intervention. ANOVA and repeated measures ANOVA were used in a number of analyses. However, these are not recommended as a general approach for longitudinal data due to the following limitations: (1) they are not able to deal with missing data, (2) they cannot model the covariance among repeated measures and (3) a repeated measures ANOVA assumes there is an exchangeable auto-correlation structure between any two observations for the same individual [37]. More complex analysis methods were employed in the SMART studies that aimed to find the optimal adaptive treatment strategy.

Trials of proportionate interventions often lead to a complex hierarchical structure of data, with hierarchical clustering introduced by both treatment or centre, in addition to the longitudinal data.

A minority of studies considered the different stages of the interventions. Some stepped-care studies used an intention-to-treat analysis to compare the intervention group to the control group after each step individually and after the whole intervention period. Only one study explicitly evaluated the effectiveness of the different components as a key objective [17]. Without consideration of the separate component parts of a proportionate intervention we assume that each component will in itself be effective. Though this may be true, the effectiveness of the components might alter as they are incorporated with one another. By design, the population size of a stepped-care trial decreases as it passes through the steps. This makes any comparisons between stages either impossible or very difficult unless the study has been designed to account for this. It is possible to evaluate the effectiveness of each stage of a proportionate intervention, as done by Lamb [17], by randomising patients who are eligible to the active or control treatment, regardless of the treatment they received at the previous stage. In certain scenarios, it would be unethical or impossible to withhold the next stage treatment of a proportionate intervention if a patient were eligible (for example, if an unstaged version of the active treatment being tested was used as the control treatment or if each stage builds upon the previous stage).

### Limitations

Due to resource limitations of this review, it was not possible to supplement the database by checking reference lists, conference proceedings or trial registries. Further work may include a supplementary search. We included only articles published in English. The studies included were mainly stepped care, which may suggest that the search criteria or eligibility criteria were unable to identify other types of studies that were also trials of proportionate interventions. We limited our review to articles published after 1 January 2010. However, this was to reflect current practice.

### Wider context

There are increasing pressures on health and social care services, limited resources and increasing health inequalities. Proportionate interventions have a role to play in the overarching goal of proportionate universalism, both in reducing health inequalities and providing care to those in need. If early-stage low-intensity interventions provide similar outcomes to more intensive interventions, then costs can be reduced and the health interventions will be less onerous for some patients and for health professionals. Increasing the intensity of a treatment does not necessarily lead to increased effectiveness [38].

Additionally, proportionate interventions fit within the overarching goals of personalised medicine: to make decisions appropriate to an individual patient, to make decisions that lead to the best outcomes for the patient, and to formalise clinical decision-making and make it evidence based. Personalised medicine aims to assign individuals to interventions based on their individual characteristics and to target interventions to patients likely to benefit. This requires evidence on what types of patients will benefit from different interventions, which is not always available [39]. In contrast, proportionate interventions can be self-correcting, with individuals failing to benefit from lower intensity interventions stepping up to more intense interventions.

The recommendation from the Marmot review that interventions follow a proportionate universalism framework has not been supported by the evidence base on how to evaluate or implement such interventions [2]. The proportionate universalism framework has been discussed in NICE guidelines [40] and NHS documents [41–43] and by charities [44] and public health authorities [45]. However, little has been written in the academic literature on how to actually implement proportional universalism in practice or how to assess the effectiveness of these interventions. This review provides examples of the types of interventions that fit into the proportionate universalism framework and the trial designs used to evaluate these at present.

### Implications and recommendations

There have been recent developments in adaptive treatment strategies, and trial designs now exist for optimal treatment strategies (SMARTs). There are also designs that evaluate the effectiveness of stepped-care treatments as a whole. Further research on how to design and analyse trials of proportionate interventions would benefit from considering when quantifying the effectiveness or the incremental effectiveness of each stage is necessary and how this may be implemented. This depends upon whether the separate stages have been evaluated in a trial before as well as the interactions between them. Is the interaction between the different components expected and of interest? Without this aspect, it may be unclear how all the components work and how they interact with one another.

Recent advances in designs of proportionate trials include just-in-time adaptive interventions. The design and framework are described in Nahum-Shani et al. [46] and Klasnja et al. [47]. This design is useful in, for example, the growing field of educational research for developing cluster-level adaptive interventions [48], or for comparing adaptive interventions embedded in a SMART [49]. Findings from trials using this framework are forthcoming and could form the basis of, or be included in, future systematic reviews on mobile health technologies.

Triallists need to account for the impact that multiple hierarchical levels (often present in proportionate interventions) have on the analysis. More complex mixed-effects models accounting for the various correlations may be necessary, including a consideration of methods for partially nested trials when clustering is present only in one arm [50, 51].

Of the 51 studies excluded based on the full texts, 27 were excluded due to a lack of, or the undefined nature of, the decision stages or rules in the intervention. This lack of clarity was occasionally due to the decision rule being based on a health professional’s opinion. However, a lack of clarity was also repeatedly due to limited information in the articles’ explanation of what the intervention actually entailed. If a trial is to provide fully useable information and a replicable intervention, it must give a clear explanation of the decision stages and rules. The readers can then understand the reasoning, and the process can be implemented either in a different setting or in a further trial. When reporting trials, it is important to follow both the relevant Consolidated Standards of Reporting Trials (CONSORT) [52] and the Template for Intervention Description and Replication (TIDieR) [53]. Both state that interventions must be reported with sufficient detail to allow replication, including how and when they were administered. This is particularly pertinent in proportionate interventions, such as stepped care, since the how and when are often multifaceted.

## Conclusion

The increasing demand on health and social care services and medicine has driven the move for proportionate universalism as well as the move towards fairer and more effective personalised medicine. Appropriate treatment and service provision according to individual need is key. Proportionate interventions aim to provide individuals with the care they require and reduce the burden of treatment on an individual whilst reserving resources for those most in need. The results of this review have identified various contexts and therapeutic areas in which trials of proportionate interventions are being designed and implemented, predominantly in the treatment of mental health disorders. The term ‘proportionate universalism’ was not used in any of the studies identified, though analogous terms were used, including the stepped-care model, adaptive treatment strategy and dynamic treatment regimen. The two key types of study designs found in this review included stepped-care studies and SMART studies. The effectiveness of the different stages was considered in a minority of studies and often only as a simple analysis using summary statistics. There is a need for a more consistent approach and further guidance on the design, analyses and reporting across trials of proportionate interventions, so that comparisons can be made.

## Additional files

Additional file 1 Completed PRISMA 2009 checklist. (PDF 201 kb)

Additional file 2 Reference list of studies included in this systematic review. (PDF 46 kb)

## Abbreviations

ANCOVA: Analysis of covariance
ANOVA: Analysis of variance
AUDIT-C: Alcohol use disorders identification test, consumption
BAI: Beck anxiety inventory
BSQ: Body sensations questionnaire
CBT: Cognitive behavioural therapy
CES-D: Epidemiologic studies depression scale
CGI: Clinical global impression
CGI-I: Clinical global impression, improvement scale
CGI-S: Clinical global impression, severity scale
CONSORT: Consolidated standards of reporting trials
COPD: Chronic obstructive pulmonary disease
CSR: Clinicians’severity rating
DSM-IV: Diagnostic and statistical manual of mental disorders
EF: External facilitator
EMT: Enhanced milieu teaching
EORTC-QLQ-C30: European Organisation for Research and Treatment of Cancer quality of life questionnaire
FAQ: Frequently asked questions
FBT: Family-based treatment
GAD-7: Generalised anxiety disorder 7
GP: General practitioner
HADS: Hospital anxiety and depression scale
HADS-A: Hospital anxiety and depression scale, anxiety
HADS-D: Hospital anxiety and depression scale, depression
ICD-10: International statistical classification of diseases and related health problems, 10th revision
IDS: Inventory of depressive symptomatology
IES: Impact of events scale
IF: Internal facilitator
IPC: Intensive parental coaching
JASP: Joint attention symbolic play engagement and regulation
K10: Kessler psychological distress scale
MASC: Multidimensional anxiety scale for children
MI: Motivational interview
MINI: Mini international neuropsychiatric interview
NHS: National health service
NICE: National institute for health and care excellence
NIHR: National institute for health research
PANSS: Positive and negative syndrome scale
PCTA: Parent–child treatment for anxiety
PHQ-9: Patient health questionnaire
PHQ-PD: Patient health questionnaire, panic disorder subscale
PHR: Public health research
PRISMA: Preferred reporting items for systematic reviews and meta-analyses
PSA: Prostate-specific antigen
PST: Problem-solving treatment
QoL: Quality of life
REP: Replicating effective programs
SCID-I: Structured clinical interview for DSM-IV
SCL-20: 20-item symptom checklist depression scale
SF-12: 12-item short form health survey
SGD: Speech-generating device
SMART: Sequential multiple assignment randomised trial
SPS: Sensory processing sensitivity
STAI: State-trait anxiety inventory
SUMD: Scale to assess unawareness of mental disorder
TIDieR: Template for intervention description and replication
TSCYC-PTS: Trauma symptom checklist for young children, post-traumatic stress subscale
WAD: Whiplash-associated disorders
WSAS: Work and social adjustment scale
Y-BOCS: Yale–brown obsessive–compulsive scale
