# Supplementary material for: Statistical design and analysis in trials of proportionate interventions: a systematic review
Source: Trials. 2019 Feb 28;20:151. doi: 10.1186/s13063-019-3206-x (PMC6396459; doi:10.1186/s13063-019-3206-x)
Supplement: Supplementary file 1 — Completed PRISMA 2009 checklist. (PDF 201 kb) [file 13063_2019_3206_MOESM1_ESM.pdf]

List of full-text studies included in systematic review

[1] Ell, K., Katon, W., Xie, B., Lee, P.-J., Kapetanovic, S., Guterman, J., Chou, C.-P.: Collaborative care management of major depression among low-income, predominantly hispanic subjects with diabetes a randomized controlled trial. Diabetes Care 33(4), 706–713 (2010)

[2] vant Veer-Tazelaar, P., Smit, F., van Hout, H., van Oppen, P., van der Horst, H., Beekman, A., van Marwijk, H.: Cost-effectiveness of a stepped care intervention to prevent depression and anxiety in late life: randomised trial. The British Journal of Psychiatry 196(4), 319–325 (2010)

[3] Braamse, A.M.J., van Meijel, B., Visser, O., van Oppen, P., Boenink, A.D., Eeltink, C., Cuijpers, P., Huijgens, P.C., Beekman, A.T.F., Dekker, J.: Distress and quality of life after autologous stem cell transplantation: a randomized clinical trial to evaluate the outcome of a web-based stepped care intervention. BMC Cancer 10 (2010)

[4] Patel, V., Weiss, H.A., Chowdhary, N., Naik, S., Pednekar, S., Chatterjee, S., De Silva, M.J., Bhat, B., Araya, R., King, M., et al.: Effectiveness of an intervention led by lay health counsellors for depressive and anxiety disorders in primary care in goa, india (manas): a cluster randomised controlled trial. The Lancet 376(9758), 2086–2095 (2010)

[5] Gilliam, C.M., Diefenbach, G.J., Whiting, S.E., Tolin, D.F.: Stepped care for obsessive-compulsive disorder: An open trial. Behaviour Research and Therapy 48(11), 1144–1149 (2010)

[6] Kay-Lambkin, F.J., Baker, A.L., McKetin, R., Lee, N.: Stepping through treatment: Reflections on an adaptive treatment strategy among methamphetamine users with depression. Drug and Alcohol Review 29(5), 475–482 (2010)

[7] Richter, D., Mickel, C., Acharya, S., Brunel, P., Militaru, C.: Aliskiren-based stepped-care treatment algorithm provides effective blood pressure control. International Journal of Clinical Practice 65(5), 613–623 (2011)

[8] Weiss, R.D., Potter, J.S., Fiellin, D.A., Byrne, M., Connery, H.S., Dickinson, W., Gardin, J., Griffin, M.L., Gourevitch, M.N., Haller, D.L., et al.: Adjunctive counseling during brief and extended buprenorphine-naloxone treatment for prescription opioid dependence: a 2-phase randomized controlled trial. Archives of General Psychiatry 68(12), 1238–1246 (2011)

[9] Mitchell, J.E., Agras, S., Crow, S., Halmi, K., Fairburn, C.G., Bryson, S., Kraemer, H.: Stepped care and cognitive–behavioural therapy for bulimia nervosa: randomised trial. The British Journal of Psychiatry 198(5), 391–397 (2011)

[10] Seekles, W., van Straten, A., Beekman, A., van Marwijk, H., Cuijpers, P.: Stepped care treatment for depression and anxiety in primary care. a randomized controlled trial. Trials 12(171) (2011)

[11] Tolin, D.F., Diefenbach, G.J., Gilliam, C.M.: Stepped care versus standard cognitive–behavioral therapy for obsessive–compulsive disorder: A preliminary study of efficacy and costs. Depression and anxiety 28(4), 314–323 (2011)

[12] van der Leeden, A.J., van Widenfelt, B.M., van der Leeden, R., Liber, J.M., Utens, E.M., Treffers, P.D.: Stepped care cognitive behavioural therapy for children with anxiety disorders: A new treatment approach. Behavioural and Cognitive Psychotherapy 39(01), 55–75 (2011)

[13] Apil, S.R., Hoencamp, E., Haffmans, P.M. Judith, Spinhoven, P.: A stepped care relapse prevention program for depression in older people: a randomized controlled trial. International Journal of Geriatric Psychiatry 27(6), 583–591 (2012)

[14] Karp, J.F., Rollman, B.L., Reynolds, C.F., Morse, J.Q., Lotrich, F., Mazumdar, S., Morone, N., Weiner, D.K.: Addressing both depression and pain in late life: the methodology of the ADAPT study. Pain Medicine 13(3), 405–418 (2012)

[15] Shortreed, S.M., Moodie, E.E.: Estimating the optimal dynamic antipsychotic treatment regime: evidence from the sequential multiple-assignment randomized clinical antipsychotic trials of intervention and effectiveness schizophrenia study. Journal of the Royal Statistical Society: Series C (Applied Statistics) 61(4), 577–599 (2012)

[16] Dozeman, E., van Marwijk, H.W., van Schaik, D.J., Smit, F., Stek, M.L., van der Horst, H.E., Bohlmeijer, E.T., Beekman, A.T.: Contradictory effects for prevention of depression and anxiety in residents in homes for the elderly: a pragmatic randomized controlled trial. International psychogeriatrics 24(08), 1242–1251 (2012)

[17] Nordin, K., Rissanen, R., Ahlgren, J., Burell, G., Fj¨allskog, M.-L., B¨orjesson, S., Arving, C.: Design of the study: How can health care help female breast cancer patients reduce their stress symptoms? A randomized intervention study with stepped-care. BMC Cancer 12(1), 167 (2012) [19] Jakicic, J.M., Tate, D.F., Lang, W., Davis, K.K., Polzien, K., Rickman, A.D., Erickson, K., Neiberg, R.H., Finkelstein, E.A.: Effect of a stepped-care intervention approach on weight loss in adults: a randomized clinical trial. JAMA 307(24), 2617–2626 (2012)

[18] Jakicic, J. M., Tate, D. F., Lang, W., Davis, K. K., Polzien, K., Rickman, A. D., Erickson, K, Neiberg, RH, Finkelstein EA. Finkelstein, E. A.:. Effect of a stepped-care intervention approach on weight loss in adults: a randomized clinical trial. JAMA, 307(24), 2617-2626 (2012)

[19] Wang, L., Rotnitzky, A., Lin, X., Millikan, R.E., Thall, P.F.: Evaluation of viable dynamic treatment regimes in a sequentially randomized trial of advanced prostate cancer. Journal of the American Statistical Association 107(498), 493–508 (2012)

[20] Pommer, A.M., Pouwer, F., Denollet, J., Pop, V.J.: Managing co-morbid depression and anxiety in primary care patients with asthma and/or chronic obstructive pulmonary disease: study protocol for a randomized controlled trial. Trials 13(1), 6 (2012)

[21] Lamb, S.E., Williams, M.A., Williamson, E.M., Gates, S., Withers, E.J., Mt-Isa, S., Ashby, D., Castelnuovo, E., Underwood, M., Cooke, M.W., Group, M.T.: Managing Injuries of the Neck Trial (MINT): a randomised controlled trial of treatments for whiplash injuries. Health Technology Assessment 16(49) (2012)

[22] Krebber, A.-M.H., Leemans, C.R., de Bree, R., van Straten, A., Smit, F., Smit, E.F., Becker, A., Eeckhout, G.M., Beekman, A.T., Cuijpers, P., et al.: Stepped care targeting psychological distress in head and neck and lung cancer patients: a randomized clinical trial. BMC Cancer 12(1), 173 (2012)

[23] Borsari, B., Hustad, J.T.P., Mastroleo, N.R., Tevyaw, T.O., Barnett, N.P., Kahler, C.W., Short, E.E., Monti, P.M.: Addressing alcohol use and problems in mandated college students: a randomized clinical trial using stepped care. Journal of Consulting and Clinical Psychology 80(6), 1062–1074 (2012)

[24] Rose, J.E., Behm, F.M.: Adapting smoking cessation treatment according to initial response to precessation nicotine patch. The American Journal of Psychiatry 170(8), 860–867 (2013)

[25] Watson, J., Crosby, H., Dale, V., Tober, G., Wu, Q., Lang, J., McGovern, R., Newbury-Birch, D., Parrott, S., Bland, J., et al.: AESOPS: a randomised controlled trial of the clinical effectiveness and cost-effectiveness of opportunistic screening and stepped care interventions for older hazardous alcohol users in primary care. Health Technology Assessment, No. 17.25 (2013)

[26] Oosterbaan, D.B., Verbraak, M.J., Terluin, B., Hoogendoorn, A.W., Peyrot, W.J., Muntingh, A., van Balkom, A.J.: Collaborative stepped care versus care as usual for common mental disorders: 8-month, cluster randomised controlled trial. The British Journal of Psychiatry 203(2), 132–139 (2013)

[27] Van Dijk, S.E., Pols, A.D., Adriaanse, M.C., Bosmans, J.E., Elders, P.J., Van Marwijk, H.W., Van Tulder, M.W.: Cost-effectiveness of a stepped-care intervention to prevent major depression in patients with type 2 diabetes mellitus and/or coronary heart disease and subthreshold depression: design of a cluster-randomized controlled trial. BMC Psychiatry 13(128) (2013)

[28] Arving, C., Thormodsen, I., Brekke, G., Mella, O., Berntsen, S., Nordin, K.: Early rehabilitation of cancer patients - a randomized controlled intervention study. BMC Cancer 13 (2013)

[29] Mattsson, S., Alfonsson, S., Carlsson, M., Nygren, P., Olsson, E., Johansson, B.: U-CARE: Internetbased stepped care with interactive support and cognitive behavioral therapy for reduction of anxiety and depressive symptoms in cancer–a clinical trial protocol. BMC Cancer 13, 414 (2013)

[30] Carels, R.A., Hoffmann, D.A., Hinman, N., Burmeister, J.M., Koball, A., Ashrafioun, L., Oehlhof, M.W., Bannon, E., Leroy, M., Darby, L.: Step-down approach to behavioural weight loss treatment: a pilot of a randomised clinical trial. Psychology & health 28(10), 1121–1134 (2013)

[31] van der Aa, H.P., Van Rens, G.H., Comijs, H.C., Bosmans, J.E., Margrain, T.H., van Nispen, R.M.: Stepped-care to prevent depression and anxiety in visually impaired older adults–design of a randomised controlled trial. BMC Psychiatry 13(209) (2013)

[32] Kasari, C., Kaiser, A., Goods, K., Nietfeld, J., Mathy, P., Landa, R., Murphy, S., Almirall, D.: Communication interventions for minimally verbal children with autism: A sequential multiple assignment randomized trial. Journal of the American Academy of Child & Adolescent Psychiatry 53(6), 635–646 (2014)

[33] Muntingh, A., van der Feltz-Cornelis, C., van Marwijk, H., Spinhoven, P., Assendelft, W., de Waal, M., Ader, H., van Balkom, A.: Effectiveness of collaborative stepped care for anxiety disorders in primary care: a pragmatic cluster randomised controlled trial. Psychotherapy and Psychosomatics 83(1), 37–44 (2014)

[34] Kilbourne, A.M., Almirall, D., Eisenberg, D., Waxmonsky, J., Goodrich, D.E., Fortney, J.C., Kirchner, J.E., Solberg, L.I., Main, D., Bauer, M.S., et al.: Protocol: Adaptive implementation of effective programs trial (adept): cluster randomized smart trial comparing a standard versus enhanced implementation strategy to improve outcomes of a mood disorders program. Implement Science 9(132) (2014)

[35] Hamall, K.M., Heard, T.R., Inder, K.J., McGill, K.M., Kay-Lambkin, F.: The Child Illness and Resilience Program (CHiRP): a study protocol of a stepped care intervention to improve the resilience and wellbeing of families living with childhood chronic illness. BMC Psychology 2(1) (2014)

[36] Gureje, O., Oladeji, B.D., Araya, R., Montgomery, A.A.: A cluster randomized clinical trial of a stepped care intervention for depression in primary care (STEPCARE)-study protocol. BMC Psychiatry 15(1), 1 (2015)

[37] Stoop, C., Nefs, G., Pommer, A., Pop, V., Pouwer, F.: Effectiveness of a stepped care intervention for anxiety and depression in people with diabetes, asthma or copd in primary care: A randomized controlled trial. Journal of affective disorders 184, 269–276 (2015)

[38] Stam, H., van der Wouden, J.C., van der Horst, H.E., Maarsingh, O.R.: Impairment reduction in older dizzy people in primary care: study protocol for a cluster randomised controlled trial. Trials 16(313) (2015)

[39] Lock, J., Le Grange, D., Agras, W.S., Fitzpatrick, K.K., Jo, B., Accurso, E., Forsberg, S., Anderson, K., Arnow, K., Stainer, M.: Can adaptive treatment improve outcomes in family-based therapy for adolescents with anorexia nervosa? feasibility and treatment effects of a multi-site treatment study. Behaviour Research and Therapy 73, 90–95 (2015)

[40] Schuurhuizen, C.S., Braamse, A.M., Beekman, A.T., Bomhof-Roordink, H., Bosmans, J.E., Cuijpers, P., Hoogendoorn, A.W., Konings, I.R., van der Linden, M.H., Neefjes, E.C., et al.: Screening and treatment of psychological distress in patients with metastatic colorectal cancer: study protocol of the tes trial. BMC Cancer 15(302) (2015)

[41] Haug, T., Nordgreen, T., Ost, L.-G., Kvale, G., Tangen, T., Andersson, G., Carlbring, P., Heiervang, ¨ E.R., Havik, O.E.: Stepped care versus face-to–face cognitive behavior therapy for panic disorder and social anxiety disorder: predictors and moderators of outcome. Behaviour Research and Therapy 71, 76–89 (2015)

[42] Salloum, A., Wang, W., Robst, J., Murphy, T.K., Scheeringa, M.S., Cohen, J.A., Storch, E.A.: Stepped care versus standard trauma-focused cognitive behavioral therapy for young children. Journal of Child Psychology and Psychiatry 57(5), 614–622 (2015)

[43] Wu, F., Laber, E.B., Lipkovich, I.A., Severus, E.: Who will benefit from antidepressants in the acute treatment of bipolar depression? A reanalysis of the STEP-BD study by Sachs et al. 2007, using Q-learning. International Journal of Bipolar Disorders 3(1), 1–11 (2015)

[44] Painter, J.T., Fortney, J.C., Gifford, A.L., Rimland, D., Monson, T., Rodriguez-Barradas, M.C., Pyne, J.M.: Cost-Effectiveness of Collaborative Care for Depression in HIV Clinics. Journal of Acquired Immune Deficiency Syndromes 70(4), 377–385 (2015)
